# Relationship between the inflammation/immune indexes and deep venous thrombosis (DVT) incidence rate following tibial plateau fractures

**DOI:** 10.1186/s13018-020-01765-9

**Published:** 2020-07-02

**Authors:** Dawei Liu, Yanbin Zhu, Wei Chen, Junyong Li, Kuo Zhao, Junzhe Zhang, Hongyu Meng, Yingze Zhang

**Affiliations:** 1 Department of Orthopaedic Surgery, Tianjin Nankai Hospital, Tianjin, 300100 P. R. China; 2 Department of Orthopaedic Surgery, The 3rd Hospital of Hebei Medical University, Shijiazhuang, 050051 Hebei P. R. China; 3 Key Laboratory of Biomechanics of Hebei Province, Shijiazhuang, 050051 Hebei P. R. China; 4 Orthopaedic Institution of Hebei Province, Shijiazhuang, 050051 Hebei P. R. China; 5 Chinese Academy of Engineering, Beijing, 100088 P. R. China

**Keywords:** Inflammatory/immune index, Biomarkers, Deep venous thrombosis, Tibial plateau fractures, Epidemiology

## Abstract

**Objective:**

To determine the relationship between inflammation/immune-based indexes and deep venous thrombosis (DVT) incidence rate following tibial plateau fractures

**Methods:**

Retrospective analysis of a prospectively collected data on patients undergoing surgeries of tibial plateau fractures between October 2014 and December 2018 was performed. Duplex ultrasonography (DUS) was routinely used to screen for preoperative DVT of bilateral lower extremities. Data on biomarkers (neutrophil, lymphocyte, monocyte, and platelet counts) at admission were collected, based on which neutrophil to lymphocyte ratio (NLR), platelet to lymphocyte ratio (PLR), monocyte/lymphocyte (MLR), and systemic immune-inflammation index (SII, neutrophil* platelet/lymphocyte) were calculated. Receiver operating characteristic (ROC) was used to determine the optimal cutoff value for each variable. Multivariate logistic regression analysis was used to evaluate the independent relationship of each biomarker or index with DVT, after adjustment for demographics, co-morbidities, and injury-related variables.

**Results:**

Among 1179 patients included, 16.3% (192/1179) of them had a preoperative DVT. Four factors were identified to be significantly associated with DVT, including open fracture, increased D-dimer level. Among the biomarkers and indexes, only platelet and neutrophil were identified to be independently associated with DVT, and the significance remained after exclusion of open fracture. The other independent variables were elevated D-dimer level (> 0.55 mg/L), male gender, and hypertension in the sensitivity analysis with open fractures excluded.

**Conclusion:**

These identified factors are conducive to the initial screening for patients at risk of DVT, individualized risk assessment, risk stratification, and accordingly, development of targeted prevention programs.

## Introduction

Tibial plateau fracture represented 1–2% of adult fractures and 32% of peri-knee fractures [1], with a population-based incidence of 10.3 per 100,000 person-years [2]. It is generally accepted that deep venous thrombosis (DVT) is a significant cause of morbidity, pulmonary embolism, and even mortality, especially in patients with trauma or undergoing major orthopedic surgery [3, 4]. The previous reports showed that 17.3 to 23.9% of patients with tibial plateau fracture would develop deep vein thrombosis (DVT) before they were operated [5, 6]. Extensive and deep understanding of the epidemiologic characteristics of DVT, particularly the relevant risk factors, was of extreme importance in prevention and management. By far, multiple risk factors associated with DVT have been identified, including advanced age, male gender, obesity, a history of DVT or pulmonary embolism, immobility, smoking, or fracture itself [7–10].

In addition to external factors, there are increasing evidences that the internal factors, namely the systemic inflammation/immune response to trauma (hip fracture) or major surgical trauma (arthroplasty), played an important role in the development of DVT. Alexandru et al. [11] found the significant change of some inflammation/immune indexes (neutrophil/lymphocyte ratio (NLR) and platelet/lymphocyte ratio (PLR)) after long-bone fracture. Moreover, researchers have identified the significant correlation of NLR, PLR, or monocyte to lymphocyte ratio (MLR) with acute DVT after major orthopedic surgery [12–14]. However, the role of these inflammation/immune indexes in the development of DVT was not consistently significant [15]. Unlike other markers of inflammation, these indexes are calculated by the readily obtained biomarkers from a hemogram with an automated differential, which are routinely measured. If these inexpensive and readily available indexes exhibit their value in predicting DVT after traumatic fractures, they will help identify patients at risk of DVT and improve the specificity in detection of DVT.

As far as we know, specified at tibial plateau fracture, there remains no relevant data on the relationship between inflammation/immune indexes and preoperative DVT occurrence. In this study, we used the prospectively collected data in a level I trauma center to address this issue. Our aims were (1) to identify the optimal cutoff values of biomarkers or their derived indexes, (2) to evaluate their predictive ability for development of DVT, and (3) to evaluate their independent relationship with DVT after adjustment for demographics, co-morbidities, and injury-related characteristics.

## Methods

Data used in this study were obtained from the database of surgical site infection in orthopedic surgery (SSIOS), in which a prospective method was used to collect data on patients who underwent orthopedic surgeries between October 1, 2014, and December 31, 2018, and surveillance of surgical site during hospitalization and telephone follow-up after discharge were conducted to identify surgical site infections. The ethics committee of the 3rd Hospital of Hebei Medical University approved the SSIOS (NO 2014-015-1), and all the participants had written the informed consent.

### Inclusion and exclusion criteria

Patients meeting the following criteria were included: age of 18 years or older, definite diagnosis of tibial plateau fracture, and complete data available. The exclusion criteria were pathological (metastatic) fracture, old fracture (> 3 weeks from injury), tibial plateau fracture combined with vascular injury, concurrent fractures in other locations, patients with lower extremity myodynamia abnormality, patients with history of DVT or other thrombotic events, or current use of anticoagulants due to chronic comorbidities.

According to our policy, all patients received basic thromboprophylaxis immediately after admission, consisting of chemical (low molecular weight heparin (LMWH), 2500–4100 IU once daily, subcutaneous injection) and elevation of the injured lower extremity for each patient.

### Diagnosis of DVT

Guideline for the diagnosis and treatment of deep vein thrombosis (3rd edition) proposed by Chinese Medical Association [16] was used to diagnose DVT. Before the operation, routine duplex 106 ultrasonography (DUS) scanning of bilateral lower extremities was performed to detect potential DVT in femoral common, superficial femoral, deep femoral, popliteal, posterior tibial, anterior tibial, and peroneal vein. The positive criteria of DUS scanning were set as noncompressibility, lumen obstruction or filling defect, lack of respiratory variation in above knee segments, and inadequate flow augmentation to calf and foot compression maneuvers [17]. Due to the less clinical significance, superficial or intermuscular vein thrombosis (soleal or gastrocnemius vein thrombosis) were not included [18, 19].

### Data collection and definition

Biomarkers or biomarker-derived inflammatory/immune indexes were obtained from hematologic tests carried out after admission and before the definite operation. These data included neutrophil, lymphocyte, monocyte, and platelet counts. The NLR was defined as the neutrophil count divided by lymphocyte count, PLR as the platelet count divided by the lymphocyte count, and MLR as monocyte count divided by lymphocyte count. The systemic immune-inflammation index (SII) was calculated as: platelet count × neutrophil count/lymphocyte count [20]. Given the importance in predictive ability or diagnosis of DVT, plasma D-dimer level was also included.

The other potential factors included demographics (age, gender, body mass index (BMI)), current cigarette and alcohol consumption, the comorbidities (hypertension, diabetes, chronic heart disease), and fracture-related factors (injury mechanism (low- or high-energy trauma), open or closed fracture, fracture classification based on Schatzker classification system).

The BMI (kg/m^2^) was divided using the criteria recommended by the Chinese working group on obesity: normal (18.5–23.9), underweight (< 18.5), overweight (24.0–27.9), and obesity (≥ 28.0) [21]. Low-energy injury was defined as an injury caused by a fall from a standing height, while fall from a height more than 2 m or motor accidents were defined as high-energy injury.

### Statistical analysis

Continuous variables were expressed by mean and standard deviation (SD). The categorical data were expressed as number and percentage (%) and were evaluated by chi-square or Fisher’s exact test, as appropriate.

For biomarker (neutrophil, lymphocyte, monocyte, and platelet counts) and biomarker-derived inflammation/immune indexes (NLR, PLR, MLR, and SII) and the plasma D-dimer level in continuous variable, we constructed receiver operating characteristic (ROC) to determine the optimal cutoff value for each variable, when Youden index (sensitivity + specificity − 1) was maximum. The significance of the ROC curve was tested using the area under the curve (AUC) analysis, with *p* < 0.05 as significance level. On basis of the cutoff values determined, each variable was divided in to two groups, and the chi-square or Fisher’s exact test was performed, as appropriate. We also constructed ROC curve and used the generated AUC to evaluate the discriminatory ability of each biomarker or inflammation/immune index, when they were in dichotomous variable.

In the multivariate logistics regression model, the included variables were those tested as statistically significant in the univariate analyses. The stepwise backward elimination method was used to exclude variables not significantly affecting the development of DVT. In the final model, variables with *p* < 0.10 were retained, and the correlation strength is indicated by odds ratio (OR) and 95% confidence interval (95% CI). The significance level was *p* < 0.05. Fitting degree of the final model was evaluated by Hosmer-lemeshow (H-L) test, and *p* > 0.05 indicated the acceptable result. SPSS23.0 was used to perform all the tests (IBM, armonk, New York, USA).

## Results

In this study, a total of 1179 patients with tibial plateau fractures were included, consisting of 742 males and 437 females, with an average of 45.6 years (Sd, 13.6; range, 18–82). The mean days from admission to operation were 4.2 ± 4.9 days (0–16 days). A total of 192 (16.3%) had a preoperative DVT. The preoperative DUS was performed at a mean of 3.9 ± 3.6 days (range 0–17 days) after injury, by 5 different technicians.

**Fig. 1:**
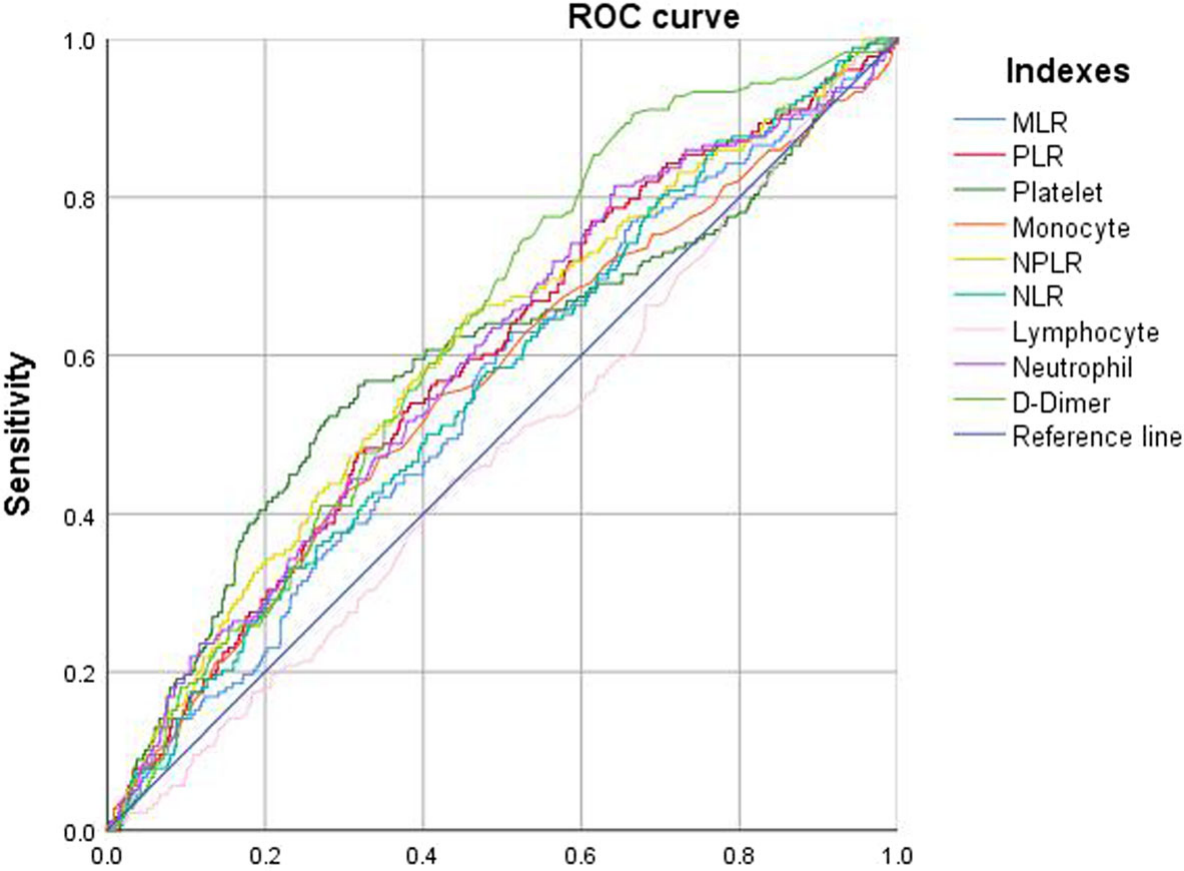
ROC to determine the optimal cutoff value for each biomarker or inflammatory/immune index, when in continuous variable

**Fig. 2:**
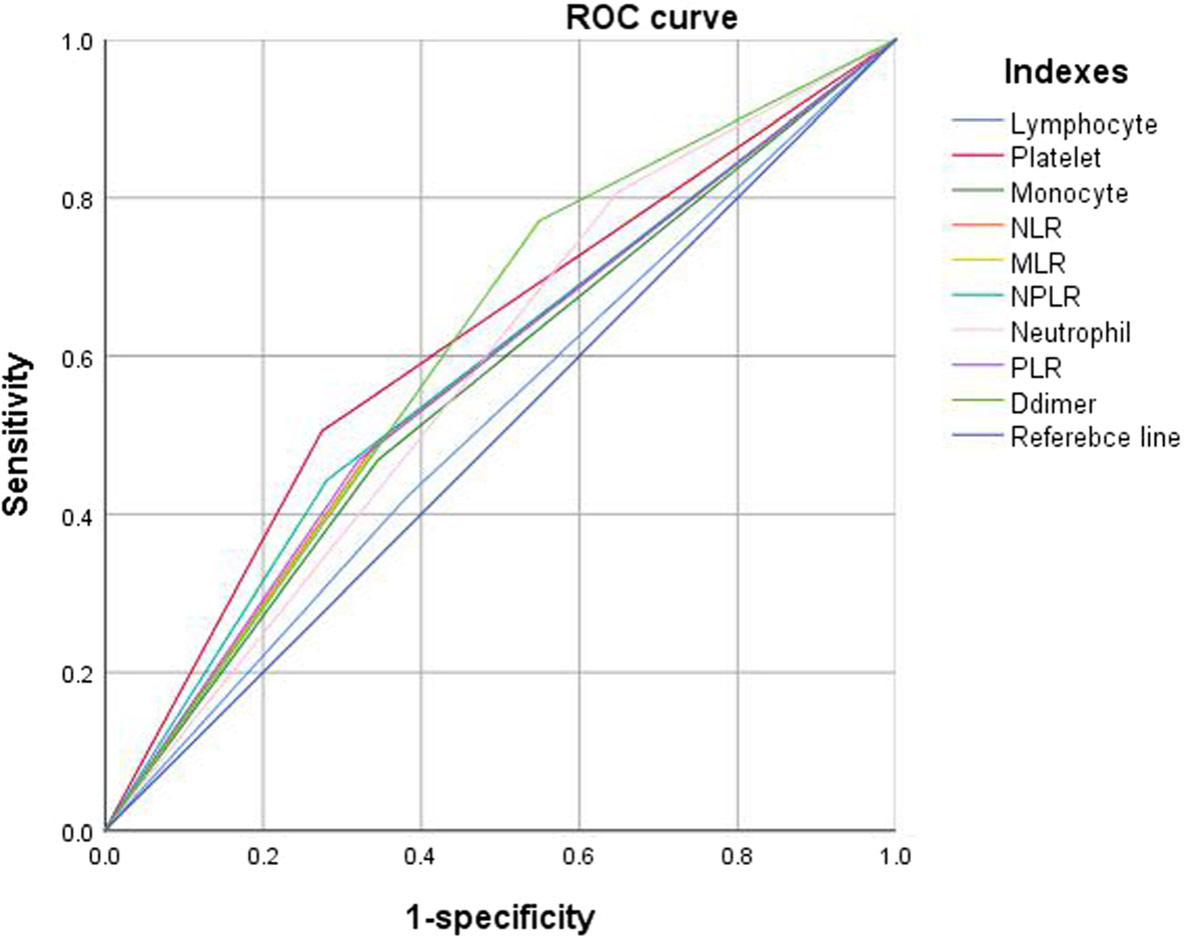
The predictive ability (sensitivity, specificity, and AUC) of each biomarker or inflammatory/immune index, when in dichotomous variable

The optimal cutoff value for each biomarker or inflammation/immune index was as follows: neutrophil count, 5.02 × 10^9^/L; lymphocyte count, 1.24 × 10^9^/L; monocyte, 0.78 × 10^9^/L; platelet count, 278 × 10^9^/L; NLR, 2.90; PLR, 207; MLR, 0.50; SII, 1066; and D-dimer, 0.55 mg/L (Fig. 1 and Table 1). When evaluated as categorical variables, the AUC showed the best discrimination ability for PLT (AUC, 0.615; 95% CI, 0.570–0.660), followed by D-dimer (AUC, 0.611; 95% CI, 0.570–0.652) (Fig. 2 and Table 2).

**Table 1:** The ROC and AUC to determine the optimal cutoff value for each index in continuous variable

Variable	Optimal cutoff value	AUC	95% CI		*p*
			Lower limit	Upper limit	
Lymphocyte	1.24 × 10^9^/L	0.481	0.435	0.527	0.422
Platelet	278 × 10^9^/L	0.598	0.548	0.648	0.000
Monocyte	0.78 × 10^9^/L	0.562	0.514	0.610	0.009
Neutrophil	5.02 × 10^9^/L	0.595	0.549	0.641	0.000
NLR	2.90	0.565	0.520	0.611	0.006
MLR	0.50	0.552	0.506	0.598	0.028
PLR	207	0.591	0.546	0.636	0.000
SII	1066	0.605	0.559	0.651	0.000
D-Dimer	0.55 mg/L	0.628	0.588	0.669	0.000

**Table 2:** The ROC and AUC to evaluate the discriminatory ability of each index in categorical variable

Variable	Sensitivity	Specificity	AUC	95% CI		*p*
				Upper limit	Lower limit	
Lymphocyte	0.422	0.618	0.520	0.475	0.565	0.381
Platelet	0.505	0.715	0.615	0.570	0.660	0.000
Monocyte	0.469	0.655	0.562	0.517	0.607	0.007
Neutrophil	0.807	0.352	0.579	0.538	0.621	0.000
NLR	0.490	0.658	0.574	0.529	0.618	0.001
MLR	0.490	0.653	0.572	0.527	0.616	0.002
PLR	0.469	0.679	0.574	0.529	0.619	0.001
SII	0.557	0.719	0.581	0.536	0.626	0.000
D-dimer	0.771	0.451	0.611	0.570	0.652	0.000

From the univariate analyses, we could find that the rate of DVT was significantly different between patients and non-DVT patients in terms of gender, prevalence of hypertension, current smoking, open fracture, fracture type based on Schartzker classification system, platelet, monocyte, neutrophile, NLR, PLR, MLR, and SII (Table 3).

**Table 3:** Univariate analyses of factors associated with preoperative DVT following tibial plateau fracture

Variables	DVTs/total (incidence)	***P***
**Gender**		0.003
**Male**	139/742 (18.7%)	
**Female**	53/437 (12.1%)	
**Age**		0.108
18–44	81/558 (14.5%)	
45–64	96/509 (18.9%)	
65 or older	15/112 (13.4%)	
**BMI (kg/m**^**2**^**)**		
18.5–23.9	44/362 (12.2%)	
< 18.5	2/21 (9.5%)	
24.0–27.9	102/511 (20.0%)	
≥ 28.0	44/286 (15.4%)	
**Diabetes mellitus**	23/154 (14.9%)	0.627
**Hypertension**	47/214 (22.0%)	0.013
**Chronic heart disease**	14/56 (25.0%)	0.070
**Fracture type (Schatzker)**		0.022
I-IV	133/793 (16.8%)	
V-VI	59/385 (15.3%)	
**Mechanism (high-energy)**	120/758(15.8%)	0.571
**Open fracture**	22/71(31.0%)	0.001
**Current smoking**	37/166 (22.3)	0.024
**Alcohol consumption**	20/107 (18.7%)	0.480
**D-Dimer** (> 0.55 mg/L**)**	148/690 (21.4%)	< 0.001
**Neutrophil count (> 5.02 × 10**^**9**^**/L)**	155/795 (19.5%)	< 0.001
**Lymphocyte (< 1.24 × 10**^**9**^**/L)**	81/458 (17.7%)	0.299
**Monocyte (> 0.78 × 10**^**9**^**/L)**	90/431 (20.9%)	0.001
**Platelet (> 278 × 10**^**9**^**/L)**	97/368 (26.4%)	< 0.001
**NLR (> 2.90)**	94/432 (21.8%)	< 0.001
**PLR (> 206)**	90/407 (22.1%)	< 0.001
**MLR (> 0.50)**	94/436 (21.6%)	< 0.001
**SII (> 1066)**	85/362 (23.5%)	< 0.001

*DVT* deep vein thrombosis, *BMI* body mass index, *NLR* neutrophil to lymphocyte rate, *PLR* platelet to lymphocyte rate, *MLR* monocyte to lymphocyte rate, *SII* systemic immune-inflammation index

In the final multivariate logistic regression model, four risk factors were identified to be associated with DVT, including open fracture, neutrophil (> 5.02 × 10^9^/L), D-dimer level (> 0.55 mg/L), and PLT > 278 × 10^9^/L (Table 4). The H-L test showed the good fitness (*X*^2^ = 2.428, *p* = 0.787; Nagelkerke *R*^2^ = 0.111).

**Table 4:** Multivariate analysis of risk factors associated with DVT, with inclusion or exclusion of open fracture

Multivariate analysis with open fracture included			Sensitivity analysis with exclusion of open fracture		
Variables	OR and 95% CI	***P***	Variables	OR and 95% CI	***P***
Open fracture (vs close)	2.30 (1.30–4.07)	0.004	Gender (male vs female)	1.71 (1.17–2.49)	0.006
D-dimer (> 0.55 mg/L)	2.20 (1.47–3.28)	< 0.001	D-dimer (> 0.55 mg/L)	2.36 (1.59–3.50)	< 0.001
Neutrophil count (> 5.02 × 10^9^/L)	1.75 (1.13–1.71)	0.012	Neutrophil count (> 5.02 × 10^9^/L)	1.59 (1.05–2.41)	0.030
Platelet count (> 278 × 10^9^/L)	2.42 (1.71–3.42)	< 0.001	Platelet count (> 278 × 10^9^/L)	2.66 (1.88–3.75)	< 0.001
			Hypertension	1.71 (1.13–2.58)	0.011

We also performed the sensitivity analysis after excluding the 71 open fractures. The results showed that neutrophil (> 5.02 × 10^9^/L), D-dimer level (> 0.55 mg/L), and PLT > 278 × 10^9^/L remained significant in the multivariate model. Also, the gender (male vs female) and hypertension were identified to be associated with occurrence of DVT (Table 4). The H-L test showed the good fitness (*X*^2^ = 5.668, *p* = 0.684; Nagelkerke *R*^2^ = 0.127).

## Discussion

This study demonstrated that several commonly used biomarker-based inflammatory/immune indexes as NLR, PLR, MLR, and SII were not independently associated with the occurrence of preoperative DVT after tibial plateau fractures. Platelet count and neutrophil count were demonstrated to be independent risk factors associated with DVT, regardless of injury type. Specified at closed tibial plateau fractures, male gender and hypertension were also identified as independent risk factors for DVT.

Over the past decade, many peripheral hemogram-derived indexes such as NLR, PLR, LMR, and SII demonstrated to be closely associated with systemic inflammation/immune response status, and be of predictive value in prognosis of various infectious, oncological, and autoimmune diseases [22–25]. The underlying mechanism might be the cascade of inflammatory cytokines and chemokines, which were initiated by inflammation dysfunctional lymphocytes, provoked neutrophil, and macrophage aggregation [26]. More recently, these inflammation/immune indexes have been increasingly used in major orthopedic surgeries and were demonstrated to be associated with injury severity or perioperative complications. Barker et al. [12] found the positive relationship between increased NLR level (day 1 and day 2, pre- and postoperative) and the risk of venous thromboembolism after total knee arthroplasty. In addition, researchers have successively reported independent association of MLR [14], NLR [27], and PLR [27] with postoperative DVT in total joint arthroplasty.

Increasing evidences have showed that inflammatory response played an important role in the development of DVT. In fact, coagulation activation and inflammation reaction were intimately related because multiple cellular factors were involved in both processes, including but not limited to monocyte, neutrophils, and platelets [28–30]. In an animal experiment, von Bruhl et al. [30] demonstrated the monocytes, neutrophils, and platelets cooperated to initiate and propagate venous thrombosis. In addition, inflammatory/immune response to trauma was the important contributing factor for development of DVT. Alexandru et al. [11] evaluated the levels of hematology panel biomarkers in 148 patients with long-bone fractures and found that patients with fractures had significantly higher NLR level, compared to controls. However, in this study, we did not demonstrate the significant relationship between any of these inflammatory/immune indexes with DVT. This might be explained by the fact that these derived indexes would have more “intersections” with the original hemogram indexes, and it was possible that algorithm between biomarkers did not exhibit the significant independent predictive effect on DVT.

In contrast, platelet and neutrophil count were found to be independently associated with DVT following tibial plateau fractures, regardless of the injury type. It is therefore suggested that increased number of neutrophils reflected the extent of inflammatory response, and activation of increased platelets is the necessity of the formation of DVT. The previous basic researches have described different mechanisms of DVT formation, including neutrophil extracellular traps (NETs), neutrophil histone modification [31–33], and trauma-induced activation of platelets and secondary cascade reaction via secretion of aggregatory mediators [34]. In this study, we also evaluate the predictive ability of them. The sensitivity neutrophil count > 5.02 × 10^9^/L was 0.807, a relatively high level, which could be used for initial screening of patients at-risk of DVT. The sensitivity of platelet count > 278 × 10^9^/L was relatively low (0.505) but with a moderate specificity of 0.715; therefore, it might be a useful auxiliary tool to exclude patients without a DVT.

D-dimer in plasma reflected the secondary increased fibrinolytic activity and the hypercoagulability, which was a well-established highly sensitive marker of thrombotic events, although the specificity was poor [35]. Another concern was the concentration of D-dimer varied in different settings. For example, D-dimer concentration increased with age; therefore, several studies suggested the use of age-adjusted cutoff values in patients with suspected DVT [36, 37]. In this study, we determined that the optimal cutoff value of D-dimer level was 0.55, slightly higher than the commonly used values, which might be associated with setting of trauma. The multivariate analysis showed that DVT > 0.55 mg/L was associated with 2.42-fold risk of DVT in overall patients, and with 2.36-fold risk in patients with closed fractures. This result demonstrated the stability of D-dimer in predicting or diagnosing DVT. We re-confirmed the low specificity D-dimer that was 0.451, higher than that of the previous studies in different settings, even with age-adjusted values used [37].

This study had some limitations. Firstly, as other multivariate analyses, not all the potential factors that affect the occurrence of DVT could be included, such as immobilisation of the injured extremity. As such, the residual confoundings remained. Secondly, C-reaction protein (CRP) was an important inflammatory biomarker predictive of DVT formation, but only a fraction of patients had the relevant data because it was not routinely measured in our hospital. Thirdly, the AUC for D-dimer, platelet, and neutrophil count was 0.628, 0.698, and 0.595, respectively, which indicated the moderate to poor predictive ability or reliability for DVT. Therefore, they should be treated cautiously, and in the current condition, combined diagnostic method with these factors may be a settlement. Fourthly, we determined the association rather than the causation between variables and DVT; therefore, these results should be interpreted with caution. Fifthly, this is a tertiary referral hospital, and patients referred would have more severe injury; therefore, the prevalence of DVT might be overestimated, and the generalizability of the results might be somewhat affected.

In summary, 16.3% of patients had preoperative DVT after tibial plateau fracture, and among the common biomarkers or biomarker-based inflammatory/immune indexes, only platelet and neutrophil were identified to be associated with development of DVT, regardless of injury pattern. These factors are conducive to the initial screening for patients at risk of DVT, individualized risk assessment, risk stratification, and accordingly development of targeted prevention programs.

## Data Availability

All the data will be available upon motivated request to the corresponding author of the present paper

## Abbreviations

DVT: Deep venous thrombosis
SSIOS: Surgical site infection in orthopedic surgery
BMI: Body mass index
DUS: Duplex ultrasonography
NLR: Neutrophil to lymphocyte ratio
PLR: Platelet to lymphocyte ratio
MLR: Monocyte to lymphocyte ratio
SII: Systemic immune-inflammation index
ROC: Receiver operating characteristic
SD: Standard deviation
OR: Odds ratio
